# Enhanced IL-6/IL-6R Signaling Promotes Growth and Malignant Properties in EBV-Infected Premalignant and Cancerous Nasopharyngeal Epithelial Cells

**DOI:** 10.1371/journal.pone.0062284

**Published:** 2013-05-01

**Authors:** Guitao Zhang, Chi Man Tsang, Wen Deng, Yim Ling Yip, Vivian Wai-Yan Lui, Sze Chuen Cesar Wong, Annie Lai-Man Cheung, Pok Man Hau, Musheng Zeng, Maria Li Lung, Honglin Chen, Kwok Wai Lo, Kenzo Takada, Sai Wah Tsao

**Affiliations:** 1 Department of Anatomy, Li Ka Shing Faculty of Medicine, The University of Hong Kong, Hong Kong, SAR, China; 2 Department of Anatomy, Histology and Embryology, Capital Medical University, Beijing, China; 3 Department of Otoloaryngology, University of Pittsburgh School of Medicine, Pittsburgh, Pennsylvania, United States of America; 4 Department of Health Technology and Informatics, Hong Kong Polytechnic University, Hong Kong, SAR, China; 5 State Key Laboratory of Oncology in Southern China, Cancer Institute, Sun Yat-sen University, Guangzhou, China; 6 Department of Clinical Oncology, Li Ka Shing Faculty of Medicine, The University of Hong Kong, Hong Kong, SAR, China; 7 Department of Microbiology, Li Ka Shing Faculty of Medicine, The University of Hong Kong, Hong Kong, SAR, China; 8 Department of Anatomical and Cellular Pathology, The Chinese University of Hong Kong, Hong Kong SAR, China; 9 Institute for Genetic Medicine, Hokkaido University, Sapporo, Japan; The Chinese University of Hong Kong, Hong Kong

## Abstract

Nasopharyngeal carcinoma (NPC) is etiologically associated with Epstein-Barr virus (EBV) infection. However, the exact role of EBV in NPC pathogenesis remains elusive. Activation of signal transducer and activator of transcription 3 (STAT3) is common in human cancers including NPC and plays an important role in the pathogenesis and progression of human cancers. Interleukin-6 (IL-6), a major inflammatory cytokine, is a potent activator of STAT3. In this study, we report that EBV-infected immortalized nasopharyngeal epithelial (NPE) cells often acquire an enhanced response to IL-6-induced STAT3 activation to promote their growth and invasive properties. Interestingly, this enhanced IL-6/STAT3 response was mediated by overexpression of IL-6 receptor (IL-6R). Furthermore, IL-6R overexpression enhanced IL-6-induced STAT3 activation in uninfected immortalized NPE cells *in vitro*, and promoted growth and tumorigenicity of EBV-positive NPC cell line (C666-1) *in vivo*. Moreover, it is shown for the first time that IL-6R was overexpressed in clinical specimens of NPC. IL-6 expression could also be strongly detected in the stromal cells of NPC and a higher circulating level of IL-6 was found in the sera of advance-staged NPC patients compared to the control subjects. Therefore, IL-6R overexpression, coupled with enhanced IL-6/STAT3 signaling may facilitate the malignant transformation of EBV-infected premalignant NPE cells into cancer cells, and enhance malignant properties of NPC cells.

## Introduction

Nasopharyngeal carcinoma (NPC) is a distinct type of head of neck cancer with high prevalence in Southeast Asia [1]. This malignancy is characterized by Epstein-Barr virus (EBV) infection, as well as its highly inflammatory stroma and metastatic nature [2]. EBV is present in virtually all cases of undifferentiated NPC in endemic regions and has long been postulated to be a crucial etiologic factor for NPC pathogenesis. Yet to date, the exact role of EBV in NPC pathogenesis remains unclear [3]–[5]. The role of inflammatory cytokines in cancer pathogenesis is well established, but their effects on EBV-infected premalignant and cancerous nasopharyngeal epithelia are poorly defined.

Cumulative evidence suggests that multiple factors are involved in NPC pathogenesis in addition to or in conjunction with EBV infection [4], [6]. STAT3 activation is common in human cancers including both hematological and solid tumors [7]. Constitutive activation of STAT3, indicated by increased expression of phospho-STAT3 (p-STAT3), has been reported in >70% of NPC specimens [8]–[10]. Under normal physiological conditions, STAT3 activation is usually transient and is tightly regulated. In tumor cells, constitutive activation of STAT3 can be caused by abnormal and sustained autocrine and/or paracrine signaling including interleukin-6 (IL-6) signaling [11]–[13]. IL-6 activates STAT3 by binding specifically to its cognate receptor on the cell membrane, i.e. IL-6R. Upon IL-6 binding, the IL-6 receptor activates the receptor-associated kinases (JAK1, JAK2 and Tyk2), which subsequently induces phosphorylation and dimerization of STAT3 (activation of STAT3). Activated STAT3 is then translocated into the nucleus to induce target gene transcription [14], [15]. Constitutive STAT3 activation promotes tumor growth and survival, as well as invasion, epithelial-mesenchymal transition and angiogenesis [16]–[18]. A recent study proposed that IL-6-mediated STAT3 activation may mediate iNOS induction in NPC, resulting in the formation of mutagenic DNA lesions which might contribute to its pathogenesis [19].

Despite the emerging biological importance of STAT3 activation in NPC, the exact mechanism of its activation remains poorly defined, especially in the context of EBV infection [20]. The L1-TR promoter of an EBV-encoded oncogene, LMP1, contains STAT3-responsive element [21]. STAT3 activation could induce transcription of LMP1 in EBV-infected NPC cells. Moreover, LMP1 expression upregulates IL-6 production [22], [23], which activates STAT3 signaling to establish a positive feedback loop of STAT3/LMP1/IL-6/STAT3 activatioin to amplify STAT3 signaling in EBV-infected NPC cells [22].

IL-6-driven STAT3 activation is of particularly significance in NPC pathogenesis. *In vivo*, the inflammatory stroma may represent a potent source of IL-6 to drive STAT3 activation in EBV-infected nasopharyngeal epithelial (NPE) cells to promote tumorigenesis and disease progression. Although IL-6 is a key cytokine mediating STAT3 signaling, the role of IL-6/STAT3 signaling in EBV-infected pre-malignant NPE cells has not been previously reported. We have previously established stable EBV infection in immortalized NPE cells [24], [25]. These EBV-infected NPE cell lines were used in this study to examine the intricate relationship between STAT3 activation and EBV infection. Interestingly, we observed for the first time that stable EBV infection in immortalized NPE cells often resulted in potentiation of their responses to IL-6-induced STAT3 activation. Furthermore, STAT3 activation in these EBV-infected NPE cells potentiated their anchorage independence and invasive properties *in vitro*. The mechanisms underlying the enhanced IL-6-induced STAT3 activation signaling in EBV-infected nasopharyngeal cells were examined in this study and its clinical relevance in NPC were explored.

## Materials and Methods

### Ethics statements

The studies involving human subjects were approved by the Institutional Review Board of the University of Hong Kong/ Hospital Authority Hong Kong West Cluster, and all subjects provided written informed consent prior to study participation. The animal investigations were approved by the Committee on the USE of Live Animals in Teaching & Research of the University of Hong Kong. Utmost effort was utilized to prevent suffering and minimize the numbers of mice required for each experiment.

### Cells and cell culture

The establishment and characterization of immortalized NPE cell line NP460hTert has been previously described [26]. Establishment of other NPE cell lines including NP550-cyclinD1-hTert, NP550-CDK4-hTert, NP361-cyclinD1-hTert will be reported separately. Stable EBV infection was achieved in these immortalized NPE cells and their culture conditions have been previously reported [24], [25]. EBV infection and cell culture of NPE cells were performed as previously reported [24]. Human NPC cell lines CNE2, C666-1, HONE1 were cultured in RPMI-1640 medium (Sigma, St Louis, MO, USA) containing 10% FBS at 5% CO_2_ at 37°C.

### Plasmids

The plasmid to express a constitutively active mutant STAT3 (pBabe-STAT3-C), the IL-6 promoter luciferase reporter construct (pGL3-IL-6-Luc), and the m67 luciferase reporter plasmid used for detection of STAT3 activation (luc-m67) were kindly provided by Dr. J.F. Bromberg, Department of Medicine, Memorial Sloan-Kettering Cancer Center, USA [27]. The pUC-IL-6R used for expression of IL-6R was kindly provided by Dr. R. Stefan (Institute of Biochemistry, Christian-Albrechts-University, Germany) [28]. The retroviral expression vector (pBabe-IL-6R) used to overexpress IL-6R was generated by inserting the IL-6R fragment from the pUC-IL-6R into the pBabe vector at the SalI site. The LMP1 expression plasmid (pcDNA 2117) which was used in a previous study [29] was a generous gift from Dr. D.P. Huang, Chinese University of Hong Kong.

### Western Blotting Analysis

Western blot analyses were carried out as described previously [26]. Nuclear extracts were prepared from culturing cells using the NE-PER Nuclear and Cytoplasmic Extraction Reagents (Thermo Scientific, Rockford, IL, USA) according to manufacturer’s instructions. The antibodies used to detect IL-6R, cyclin D1 and β-actin were purchased from Santa Cruz Biotechnology (Santa Cruz, CA, USA). The antibodies against STAT3, p-STAT3 (Tyr705) and Bcl-2 were purchased from Cell signaling (Danvers, MA, USA). The antibodies against c-myc, anti-Flag were purchased from Sigma. The antibody against LMP-1 was purchased from Dako (Glostrup, Denmark). The secondary antibodies used were horseradish peroxidase-linked antibodies purchased from Invitrogen (Carlsbad, CA, USA). Detection of the antigen in Western blotting was by ECL Plus chemiluminescence reagents (GE Healthcare, Buckinghamshire, UK) according to manufacturer’s instruction.

### Electrophoretic-mobility shift assay (EMSA)

EMSA was performed according to manufactor’s instruction using a commercially available kit for STAT3 activation (Thermo Scientific). Briefly, whole cell extracts (4 µg protein) prepared from IL-6-treated and untreated cells were incubated with biotin-labeled high-affinity serum inducible element (hSIE) duplex oligonucleotide (GATCCATTTCCCGTAAATC). After gel electrophoresis, the bound STAT3:oligonucleotide was electroblotted at 350 mA to Biodyne B membrances (nylon) and fixed using a UV crosslinker. The membranes with the bound oligonucleotide transferred were blocked, labeled with streptavidin-HPR conjugate at 1∶1000 concentration, and exposed to ECL Plus chemiluminescence reagents (GE Healthcare) according to manufacturer’s instruction. Supershifting of the STAT3:oligonucleotide complexes were detected after 30 min preincubation with anti-STAT3 antibody (Santa Cruz).

### Transwell Invasion assay

The Boyden chamber invasion assay was performed using Transwell chamber from the Milipore Company (6.4 mm in diameter with 8.0 µm pore size) according to a previously published method [30]. Cell migration was determined by averaging the number of cells migrated in ten randomly chosen microscopic fields at ×400 magnification. Data presented are the mean ± SD of triplicate wells.

### Transient plasmid transfection and Luciferase reporter assay

Lipofectamine^™^ 2000 (Invitrogen) was used for transient plasmid DNA transfection according to the manufacturer’s instruction. For measurement of STAT3 transactivating activity in HONE1 and HONE1-EBV cells, 0.8 µg of m67 luciferase reporter (luc-m67) plasmid per sample was used. Detection of STAT3 activation was performed with co-transfection of RcCMV STAT3-C or control vector with the luciferase reporter plasmid. For measuring IL-6 gene promoter activity in HONE1 cells, 0.8 µg of pGL3-IL-6-Luc per sample was used. The ratio of firefly luciferase reporter construct and Renilla used was 200:1. Thirty six hours after transfection, cells were lysed, and the luciferase activities were measured using Dual-Luciferase Reporter Kit (Promega, Madison, WI, USA). The relative luciferase activity was normalized against the value of Renilla luciferase signal.

### Real-time Quantitative PCR

RNA was isolated from samples using TRIzol (Invitrogen). RNA was reversely transcribed to cDNA using SuperScriptII reverse transcriptase (Invitrogen) according to previously published protocols [26]. The probes and primers were designed using the Universal Probe Library system (Roche Applied Science, Mannheim, Germany) according to the manufacturer’s instruction. The reaction mixture consisted of 0.4 µl forward primer (10 µM), 0.4 µl backward primer (10 µM), 10 µl LightCycler master mix, 4 µl autoclaved Milli-Q water, 0.2 µl probe and 5 µl cDNA. Real time PCR was performed in My IQTM2 Real Time PCR Detection System (Bio-Rad, Hercules, CA, USA). Gene specific primers and probes used for the study are listed in Table 1. The relative levels of mRNA transcripts of the genes examined were normalized to levels of internal control transcript of GAPDH.

**Table 1:** Oligonucleotide primers and probes for real-time PCR.

Gene	Oligonucleotide Primer	Probe #
LMP1	5′ CCCACTCTGCTCTCAAAACC	10
	3′ GTCCTGTGGGCCATTGTC	
IL-6 receptor	5′ CACATTCCTGGTTGCTGGA	82
	3′ CAGCTTCCACGTCTTCTTGA	
IL-6	5′ GATGAGTACAAAAGTCCTGATCCA	40
	3′ CTGCAGCCACTGGTTCTGT	
GAPDH	5′ AGCCACATCGCTCAGACAC	60
	3′ GCCCAATACGACCAAATCC	

### Anchorage independent growth

Soft-agar colony assay was used to determine the anchorage independent growth of the EBV-infected and uninfected cells. The bottom agar (0.6%) was prepared by mixing 6% bacto-agar and medium at 1∶9 ratio. Two ml of upper agar (0.3%) mixed with 1×10^5^ cells were then plated on top of the bottom agar. The sizes of colonies were scored after 3 weeks incubation. Only colonies >0.2 mm in diameter were counted. Data presented are the mean ± SD of triplicate wells.

### Tumorigenicity assay in nude mice

C666-1 cells (1×10^6^ cells) ectopically expressing IL-6R were harvested and suspended in 50 µl of PBS, and then mixed with an equal volume of Matrigel^™^Basement Membrane Matrix (BD Biosciences, San Jose, CA, USA). The total volume of 100 µl cell suspension was injected into the flank of 6–8 week old male nude mice. Once tumor growth was established, tumor sizes were measured every other day. Tumor volume (mean ± SD) was calculated as length × width^2^/2 [31].

### MTT assay

Briefly, 1×10^3^ cells per well were seeded in 96-well plate and incubated overnight. Subsequently, the culture media were replaced with RPMI medium containing 1% FBS with or without recombinant human IL-6 and incubated further for the indicated time points. Thereafter, 20 µl of MTT labeling reagent (5 mg/ml in PBS; Sigma) was added to each well and incubated for 4 hr at 37°C followed by addition of 200 µl of DMSO to dissolve the formazan crystals. The absorbance was measured at 570 nm using a multiplate reader (Merck Eurolab, Dietikon, Switzerland). Each data point represented the mean ± SD of three wells per treatment group.

### Immunohistochemistry

Tissue microarray (TMA) containing control and NPC tissue specimens were obtained from NPC tissue bank established by the Center for Nasopharyngeal Carcinoma Research (RGC funded Area of Excellence Theme). Sections for immunohistochemistry were dewaxed in xylene and rehydrated in graded alcohol. Endogenous peroxidase activity was blocked by incubating slides with 3% hydrogen peroxide for 10 min. For antigen retrieval, all slides were incubated with 10 mmol/L citrate buffer (pH 6.0) for 93°C for 10 min, and then cooled down to room temperature. After that, the sections were rinsed with PBS and treated with normal blocking serum (Vector Laboratories, Inc., Burlingame, CA, USA) for 30 min. Anti-human IL-6 antibody and anti-human IL-6R antibodies (Santa Cruz) diluted in PBS (1∶100) were applied to the sections and incubated at 4°C overnight. After rinsing, all sections were further incubated for 1 h with biotin-conjugated secondary antibody and horseradish peroxidase conjugated streptavidin followed by a chromogen, diaminobenzidine (Dako). Counterstaining was performed by hematoxylin before dehydration and mounting. The negative controls were performed by adding blocking peptide to replace binding of the primary antibodies to the antigens.

### ELISA for IL-6

The circulating concentrations of IL-6 in the sera of controls and NPC patients and the supernatants of cell lines were quantitated using ELISA assay for IL-6 (R&D Systems, Minneapolis, MN, USA) according to manufacturer instructions. Duplicated samples were estimated and the averaged values were used in the analysis.

### Statistical analysis

The data from each experiment were expressed as mean ± standard derivation (SD). Student’s *t* test was used to assess the differences between experimental groups. A *p* value <0.05 was considered as statistically significant throughout this study.

## Results

### EBV-infection of immortalized NPE cells enhanced their responses to STAT3 activation induced by IL-6

**Figure 1:**
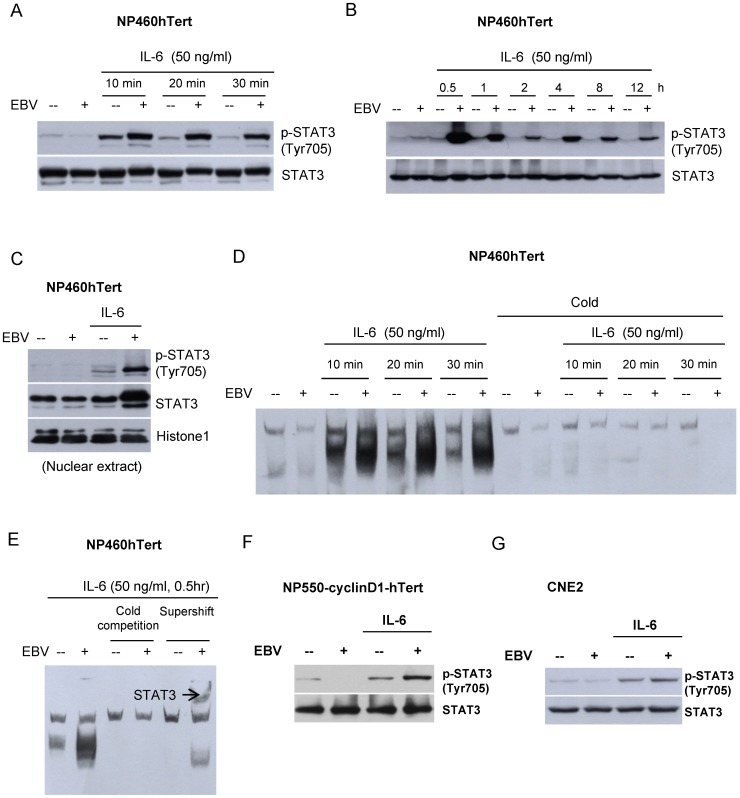
Potentiation of IL-6-induced STAT3 activation in EBV-infected NPE cells. EBV-infected and uninfected NP460hTert cells were treated with IL-6 at 50 ng/ml for (A) 10, 20 or 30 minutes and for (B) 0.5, 1, 2, 4, 8 or 12 hours. Whole cell lysates were prepared and expression of p-STAT3 (Tyr 705) was analyzed by western blot. Total STAT3 was detected as the control for protein loading. (C) Nuclear extracts were prepared from EBV-infected and uninfected NP460hTert cells with or without IL-6 treatment (50 ng/ml for 30 minutes) and subjected to Western blot analysis for p-STAT3 expression. Histone 1 was detected as the control for nuclear extract loading. (D) Whole cell protein lysates were prepared following treatment with IL-6 for the indicated time and were then subjected to EMSA analysis using biotin-labeled hSIE probe (containing STAT DNA binding elements). For “cold” competition, extracts were preincubated with unlabeled hSIE probe at 200-fold molar excess for 20 minutes before analysis. (E) The supershift assay was performed by incubating the extract with anti-STAT3 antibody for 30 minutes before EMSA analysis. The STAT3 specific supershifted complex was observed which confirmed the specificity of the EMSA for enhanced STAT3 activation in EBV-infected NP460hTert to IL-6 stimulation. (F) NP550-cyclinD1-hTert and EBV-infected NP550hTert-cyclinD1 were either treated or untreated with IL-6 at a final concentration 50 ng/ml for 30 minutes. The expression of p-STAT3 (Tyr 705) was analyzed by Western blotting. STAT3 expression was probed as a loading control of proteins. (G) CNE2 and EBV-infected CNE2 cells were either treated or untreated with IL-6 at a final concentration 50 ng/ml for 30 minutes. The expression of p-STAT3 (Tyr 705) was analyzed by Western blotting. STAT3 expression was probed as a loading control of proteins from different cell populations.

We have previously reported the establishment of stable EBV infection in a telomerase-immortalized NPE cell line (NP460hTert) [24]. When examined for responses to IL-6, we observed that the EBV-infected NP460 (NP460hTert-EBV) cells consistently displayed a much higher level of p-STAT3 (Tyr 705) compared to uninfected NP460hTert cells upon IL-6 exposure (Figure 1A). We were also able to show a sustained induction of p-STAT3 at prolonged time points after IL-6 treatment (Figure 1B). The p-STAT3 could be detected up to 12 hr in EBV-infected cells (Figure 1B). In control uninfected cells, the level of p-STAT3 already returned to basal level at 0.5 hour (Figure 1A and B). This observation further supports that IL-6-induced STAT3 activation is much more potentiated in EBV-infected cells compared to uninfected ones. We were able to confirm the enhanced activation of STAT3 to IL-6 treatment in NP460hTert-EBV cells by nuclear translocation of p-STAT3 (Figure 1C), indicating hyperactivation of STAT3 by IL-6 in EBV-infected NPE cells, but not the EBV-negative counterpart. This enhanced activation of STAT3 by IL-6 treatment in NP460hTert-EBV cells was further confirmed by EMSA (Figure 1D). The specificity of the EMSA for STAT3 activation was confirmed by supershifting the STAT3/DNA complex after binding to specific antibody to STAT3 (Figure 1E). The enhancement of IL-6-induced STAT3 activation was also observed in another immortalized NPE cell line, NP550-cyclinD1-hTert (recently immortalized by combined action of hTert and cyclin D1; manuscript in preparation) (Figure 1F). An enhanced STAT3 activation was also observed in an EBV-infected NPC cell line, CNE2, despite to a lesser extent (Figure 1G) when compared to that of immortalized NPE cell lines. The higher level of p-STAT3 in cancer cells after the IL-6 treatment might account for a weaker response to enhanced STAT3 activation after EBV infection. This weaker response in EBV-infected CNE2 was demonstrated by repeated experiments. Collectively, in the presence of EBV infection (both EBV-infected NPE and EBV-infected NPC cells), IL-6 induces hyperactivation of STAT3.

### IL-6R overexpression is involved in the potentiation of IL-6-mediated STAT3 activation in EBV-infected immortalized NPE cells

**Figure 2:**
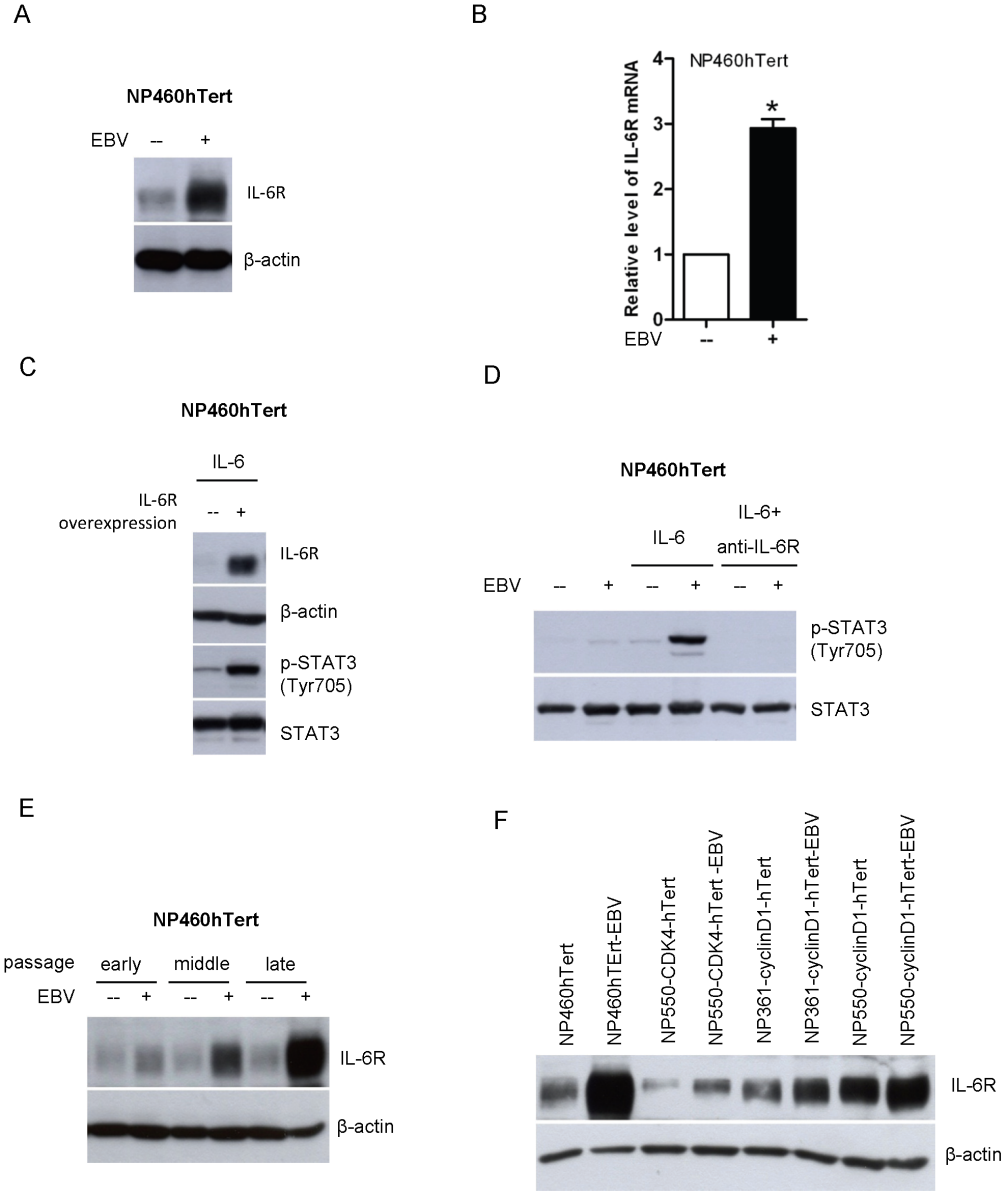
Upregulation of IL-6R in EBV-infected NPE cells is responsible for the enhanced responsiveness to IL-6-induced STAT3 activation. (A) The expression of IL-6R in EBV-infected and uninfected NP460hTert cells was analyzed by Western blot. Immunoblotting for β-actin was provided as protein loading control. (B) Total RNA was extracted and the expression levels of IL-6R mRNA in NP460hTert and NP460hTert-EBV cells were analyzed by quantitative real time RT-PCR. The mRNA levels of IL-6Rα were normalized to cellular GAPDH mRNA. Data were collected from triplicate separate experiments. * *p*<*0.05*. (C) NP460hTert cells were infected with retroviral expression vectors, pBabe-IL-6Rα or pBabe empty vectors. Stably transduced cells were treated with IL-6 at 50 ng/ml for 30 minutes. Cell lysates were prepared and examined for expression of IL-6Rα and p-STAT3 (Tyr 705) by Western blot. Immunoblottings for STAT3 and β-actin are shown as controls for protein loading. (D) NP460hTert and NP460hTert-EBV cells were pre-treated with anti-IL-6R antibody at 5 µg/ml for 30 minutes before IL-6 treatment. The expression of p-STAT3 was analyzed by Western blot. Immunoblotting for total levels of STAT3 protein is shown as controls for protein loading. (E) After EBV infection, the EBV-infected NP460hTert cells and its uninfected parental cells were continuously subcultured. Cells lysates were prepared from the cells that had been passaged for 56, 99 and 133 times (designated as early, middle and late passage). The expression of IL-6R was analyzed by Western blotting. Immunoblotting for β-actin was included as control for protein loading. (F) The levels of IL-6R expression in cells were detected by Western blot in several paired uninfected and EBV-infected cell lines, including EBV-infected and non-infected pairs of NP460hTert, NP550-CDK4-hTert, NP361-cyclinD1-hTert, NP550-cyclinD1-hTert. β-actin expressions were shown as protein loading control.

Next, we examined the underlying mechanism for such an enhanced response of EBV-infected NPE cells to IL-6. As IL-6 conveys signaling via the cell surface direct interaction with the IL-6R, we examined the expression of IL-6R in EBV-infected NPE cells and the uninfected counterparts. Unexpectedly, overexpression of IL-6R protein as well as increased levels of IL-6R mRNA transcripts were detected by Western blotting and real-time PCR, respectively, in NP460hTert-EBV cells (Figure 2A & 2B). Interestingly, the protein level of IL-6R was not directly proportionally to its transcript level. However, the protein level of a particular gene may not be completely dependent on transcriptional level. The expression level of a particular protein may also be regulated by post-translational degradation. In addition, overexpression of IL-6R by retroviral gene transfer in NP460hTert cells also conferred the enhanced responsiveness to IL-6-induced STAT3 activation (Figure 2C). Moreover, the enhanced STAT3 activation by IL-6 in NP460-EBV cells could be neutralized by anti-IL-6R antibody treatment (Figure 2D). All these results indicated that the IL-6R overexpression in NP460hTert-EBV cells play a key role in mediating the enhanced responsiveness to STAT3 activation to IL-6 treatment.

We then sought to examine whether IL-6R overexpression was a direct consequence of EBV infection or the result of progressive selection of EBV-infected NP460hTert cells overexpressing IL-6R upon prolonged passages. The expression levels of IL-6R at different passages of EBV-infected and uninfected NP460hTert cells were compared. We observed a very moderate increase in IL-6R expression at the early passage of EBV-infected NP460hTert cells, and there was a gradual increase in IL-6R expression in NP460hTert-EBV cells upon prolonged culture (Figure 2E). Note that such a change of IL-6R levels was not observed in the uninfected NP460hTert cells over prolonged cultures (Figure 2E). This strongly indicated that overexpression of IL-6R has selective growth advantage for EBV-infected NP460hTert cells and was actively selected in culture. Overexpression of IL-6R was also detected in three other stably EBV-infected immortalized NPE cell lines recently established in our laboratory by combined actions of telomerase with CDK4 or cyclin D1 (NP550-CDK4-hTert-EBV; NP361-cyclinD1-hTert-EBV; NP550-cyclinD1-hTert-EBV) [25], but not in uninfected counterparts (NP550-CDK4-hTert; NP361-cyclinD1-hTert; NP550-cyclinD1-hTert) (Figure 2F). Detailed characteristics of these newly immortalized NPE cell lines will be published separately. These observations suggested that overexpression of IL-6R has selective growth advantage in EBV-infected NPE cells and is actively selected over prolonged passages.

### Constitutive activation of STAT3 potentiates growth and malignant properties of EBV-infected cells

**Figure 3:**
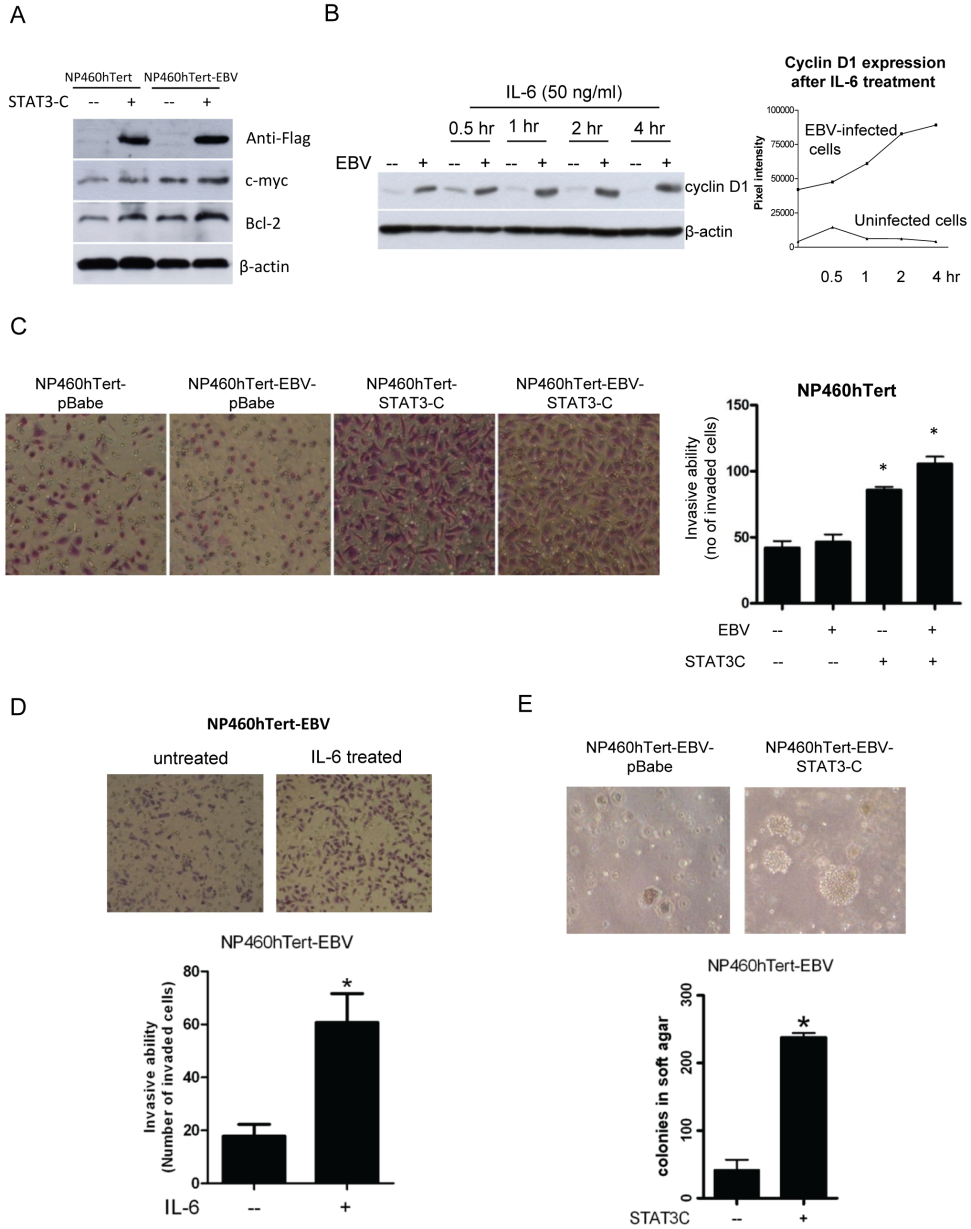
Activation of IL-6/IL-6R/STAT3 pathway conferred the malignant properties in EBV-infected NP460hTert cells. (A) c-Myc and Bcl-2 were induced by STAT3-C expression in NP460hTert and NP460hTert-EBV cells. The FLAG-tagged STAT3-C was transduced and selected in NP460hTert and NP460hTert-EBV cells. Western blotting analysis revealed the expression of FLAG indicating successful expression of STAT3C. Expression of c-myc and Bcl-2 was induced in both NP460hTert and NP460hTert-EBV cells after STAT3-C expression. (B) Enhanced expression of cyclin D1 in IL-6-treated NP460hTert-EBV cells was observed compared to control NP460hTert cells. Cells were treated or untreated with IL-6 at a final concentration 50 ng/ml for the indicated time. In the left panel, expression of cyclin D1 was analyzed by Western blot for protein expression. In the right panel, densitometry was used to quantify the expression of cyclin D1 with reference to actin. Prolonged expression of cyclin D1 was observed in IL-6-treated EBV-infected cells but not in the IL-6-treated NP460hTert cells. (C) STAT3-C-expressing NP460hTert and NP460hTert-EBV cells showed enhanced invasiveness compared with vector control cells. The invasive ability was measured by counting the number of cells that traversed the Matrigel-coated membrane in 24 hours. Data presented are the mean ± SD of triplicate wells from triplicate experiments. *, ^#^ *p*<0.05. (D) IL-6 treatment enhanced cell invasion of NP460hTert-EBV cells *in vitro*. NP460hTert-EBV cells were loaded into the upper invasion chamber with or without treatment of IL-6 (50 ng/ml). After 24 hours, the cells penetrating into the lower chamber were stained and counted. Data presented are the mean ± SD of triplicate wells from triplicate experiments. * *p*<0.05. (E) pBabe vector control- and STAT3-C-expressing cells (1×10^5^) were plated in soft agar. Three weeks later, pictures of the colonies were taken. Colonies with diameter (≥0.2 mm) were counted. The numbers of colonies shown are the means ± SD from triplicate results. * *p*<0.05

We sought to explore some of biological importance of this active selection of IL-6R expression in EBV-infected NPE cells. One hypothesis is that EBV infection may induce stress to immortalized NPE cells [5] and STAT3 activation may overcome the stress-induced growth arrest in NPE cells after EBV infection and promote their survival. Activation of STAT3 is known to activate an array of downstream target events to potentiate growth and malignant properties of cancer cells [16]. We therefore examined if STAT3 activation may potentiate growth and malignant properties of EBV-infected NPE cells. A dominant active mutant form of STAT3 (STAT3C) was transfected into EBV-infected and uninfected NP460hTert cells so as to achieve constitutive activation of STAT3 (Figure 3A). We were able to observe a moderate increase in expression of c-myc and Bcl-2, under the constitutive activation of STAT3 in EBV-infected cells compared to control uninfected cells (Figure 3A). IL-6 treatment also sustained a longer and stronger expression of cyclin D1 in EBV-infected cells (Figure 3B). Densitometry was used to analysis the cyclin D1 levels at 0.5, 1, 2, 4 hour(s) in EBV-infected cells and uninfected cells after IL-6 treatment. The cyclin D1 level was upregulated in IL-6-treated EBV-infected cells by 2 folds at 4 hour time point (Figure 3B). In contrast, the cyclin D1 levels in IL-6-treated non-infected cells only marginally upregulated at 0.5 hour and ceased to increase at other time points (Figure 3B). This data suggests that IL-6 could differentially elevate the expression of cyclin D1 in EBV-infected cells, but not in non-infected cells. Interestingly, constitutive activation of STAT3 by STAT3C expression also enhanced the invasive properties of NP460hTert-EBV compared with control uninfected NP460hTert cells as assayed by Matrigel invasion assay (Figure 3C). Furthermore, IL-6 treatment also enhanced the invasive properties of NP460hTert-EBV cells (Figure 3D). Constitutive activation of STAT3 by STAT3C expression in NP460hTert-EBV cells also induced robust anchorage independent growth in soft agar, suggesting a role of STAT3 activation to promote tumorigenic transformation of EBV-infected immortalized NPE cells (Figure 3E). The numbers as well as sizes of the anchorage independent colonies in STAT3C-expressing NP460hTert-EBV cells were increased compared to control cells infected with the empty pBabe vector (Figure 3E). All these observations showed that STAT3 activation enhances growth properties in EBV-infected immortalized NPE cells, which may explain the selection of IL-6R overexpression phenotype in EBV-infected NPE cells rendering them more responsive to IL-6-induced STAT3 activation.

### IL-6 upregulates LMP1 expression in EBV-infected NPE and NPC cells

**Figure 4:**
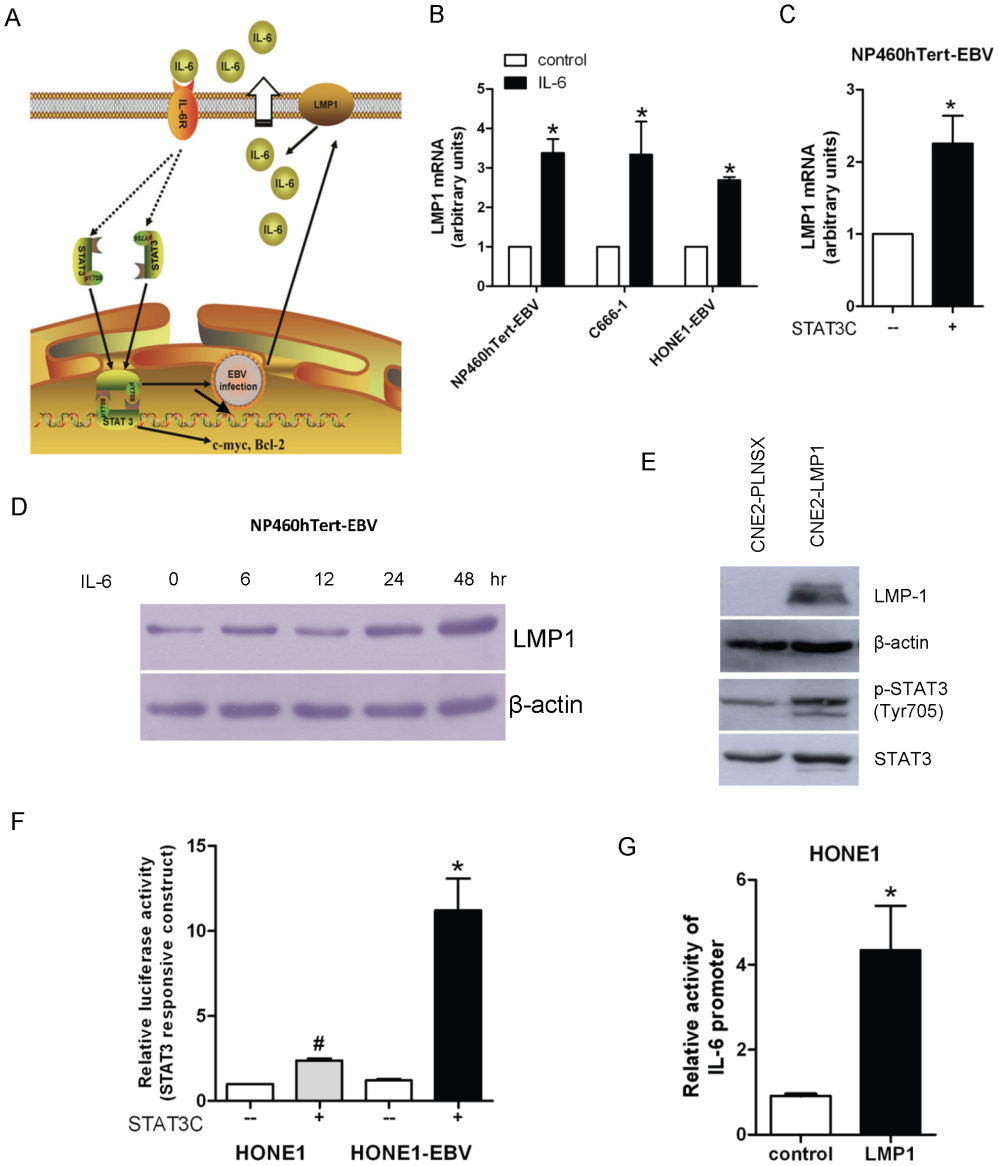
Intricate relationship between IL-6, STAT3 activation and LMP1 in EBV-infected NPE cells. (A) A schematic diagram showing the positive feedback loop of IL-6 activation of STAT3 and LMP1 expression in EBV-infected cell. (B) NP460hTert-EBV, C666-1 and HONE-EBV cells were treated with IL-6 for 4 hours. Total RNA were extracted from treated and untreated cells and examined for mRNA expression levels of LMP1 by quantitative real-time (RT-PCR). Results were normalized to GAPDH. These results were computed from triplicates of individual experiments. * *p*<0.05. (C) EBV-infected NP460hTert cells were stably transduced with STAT3-C or control vector. The mRNA expression levels of LMP1 were analyzed by quantitative RT-PCR. Results were normalized to GAPDH. * *p*<*0.05*. (D) LMP1 expression was upregulated in NP460hTert-EBV cells after treating with IL-6 for 48 hours. (E) PLNSX-LMP1 vector and PLNSX control vector were introduced into CNE2 cells by retroviral infection. Both LMP1 expression and STAT3 phosphorylation in CNE2 cells were confirmed by Western blotting. (F) HONE1 and EBV-infected HONE1 cells were transiently co-transfected with m67 firefly luciferase reporter construct (luc-m67) in conjunction with a Renilla luciferase vector and STAT3-C plasmid or control plasmid. Cell lysates were assessed for luciferase activity 36 hours after transfection. Relative luciferase activity was calculated by normalizing the firefly luciferase activity to Renilla luciferase activity. These results were computed from triplicate separate experiments. * *p*<0.05 ^#^ *p*<0.05. (G) HONE1 cells were transiently co-transfected with IL-6 firefly luciferase reporter construct (pGL3-IL-6-Luc) in conjunction with a Renilla luciferase vector and pcDNA3/2117-LMP1 or control vector. These results were computed from triplicate separate experiments. * *p*<0.05.

We have previously reported that STAT3 activation could upregulate LMP1 expression in EBV-infected epithelial cells [21], [22]. Upregulation of LMP1 increases IL-6 production to enhance STAT3 activation, hence forming a positive feed-back loop involving IL-6, STAT3 and LMP1 in EBV-infected epithelial cells (Figure 4A). This positive feedback loop may be involved in the enhanced growth and malignant properties in EBV-infected NP460hTert cells after IL-6 treatment or constitutive activation of STAT3. We confirmed that this positive-feed-back loop exists in EBV-infected cells. IL-6 treatment of NP460hTert-EBV cells (EBV-infected NPE cells), C666-1 (EBV-positive NPC cells) and HONE-EBV (EBV-reinfected NPC cells) all resulted in upregulation of LMP1 transcription (Figure 4B). Furthermore, constitutive activation of STAT3 in EBV-infected NP460hTert cells upregulated LMP1 transcription (Figure 4C). Increased LMP1 expression in NP460hTert-EBV cells could also be observed after prolonged (48 hours) treatment with IL-6 (Figure 4D). We have previously reported stable expression of LMP1 induced STAT3 activation in NPC cells, which was confirmed in this study using CNE2 cells, an NPC cell line (Figure 4E). HONE-1 cells were amenable to gene transfection and therefore used for further experiments in this part of study. We have examined and compared STAT3 activity in EBV-infected or uninfected-HONE-1 cells after transfection with the dominant active STAT3 mutant (STAT3C) using a luciferase reporter construct (pLUC-m67) to detect STAT3 activation (Figure 4F). Transfection of STAT3C stimulated a more robust activation of STAT3 in EBV-infected HONE-1 cells compared to uninfected HONE-1 cells (Figure 4F). Furthermore, LMP1 transfection into HONE-1 cells also upregulated the promoter activity of IL-6 (Figure 4G). These observations confirmed the existence of the positive feedback loop of STAT3/LMP1/IL-6/STAT3 in EBV-infected NPE cells, which may contribute to the enhancement of growth and malignant properties of EBV-infected cells.

### IL-6R overexpression promotes growth of immortalized NPE cells in vitro and tumorigenic properties of NPC cells *in vivo*

**Figure 5:**
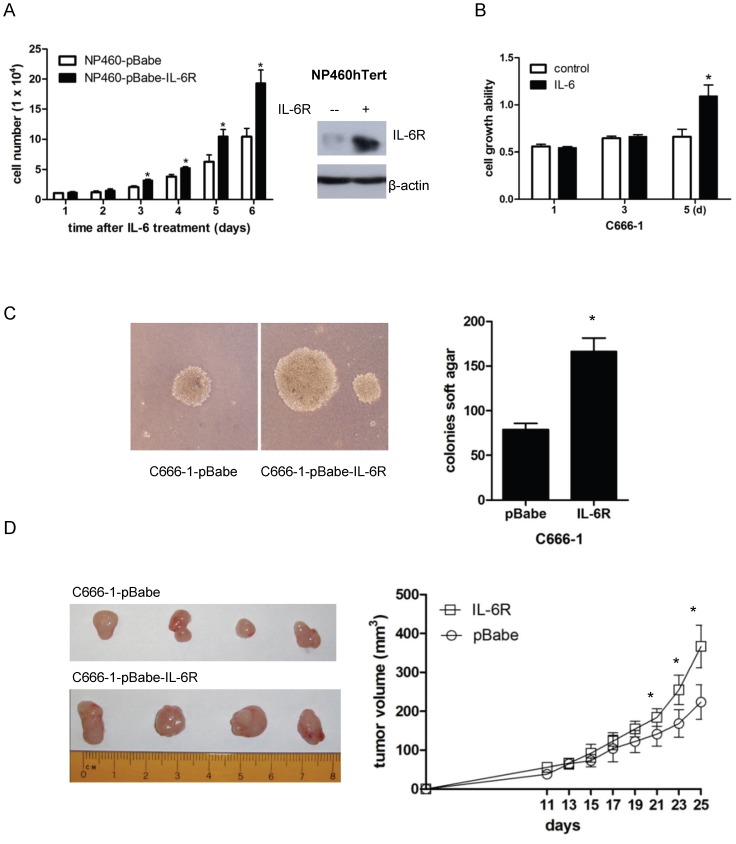
IL-6R overexpression promotes growth of immortalized NPE cells *in vitro* and tumorigenic properties of NPC cells *in vivo.* (A) pBabe control or pBabe-IL-6Rα expressing NP460hTert cells (1×10^4^ per sample) were cultured in the presence of IL-6. Cells were counted at the indicated time points. Data represent the mean of triple independent experiments ± SD. * *p*<0.05. The right panel shows the Western blot analysis of the expression of IL-6R in the NP460-pBabe-IL-6R cells and the control NP460-pBabe cells. (B) Effect of IL-6 on the growth of C666-1 cells. C666-1 cells were treated with or without IL-6 at 50 ng/ml. The MTT assay was conducted on the indicated time intervals. Each datum point represents the average of triplicate experiments. * *p*<0.05. (C) C666-1 cells were transduced with pBabe-IL-6R or pBabe by retroviral gene transfer. Vector- and IL-6R-expressing C666-1 cells were examined for colony formation in soft agar. Representative images of the soft agar colonies were shown. The number of colonies presented is mean ± SD from triplicate results. The difference in the number of soft agar colonies between pBabe- and IL-6R-expressing C666-1 cells is statistically significant (* *p*<0.05). (D) pBabe vector control- and IL-6R-expressing C666-1 cells (1×10^6^ per mouse) were subcutaneously injected into nude mice. Tumor size was measured at the indicated time points. Each time point of growth curve was calculated as the mean ± SD of five tumors per experimental group. The differences in the growth rate in xenografts established from control- and IL-6R-expressing C666-1 cells were statistically significant after injecting the cells for 21 days (* *p*<0.05).

Considering the importance of IL-6 signaling in enhancing cell growth, we postulated that expression of IL-6R, which mediates IL-6-induced STAT3 activation, should also enhance growth of immortalized NPE cells and NPC cells. As expected, IL-6 treatment promoted growth in IL-6R-overexpressing NP460hTert cells (Figure 5A). IL-6 could also enhance the growth of C666-1 cells (an EBV-positive NPC cell line) *in vitro* (Figure 5B).

We further examined the effects of IL-6R overexpression on the tumorigenicity of an EBV-positive NPC cell line, C666-1. Overexpression of IL-6R enhanced the anchorage independent growth of C666-1 cells by approximately two folds compared to control C666-1 cells infected with empty vector (Figure 5C). The sizes of the anchorage independent colonies formed were also larger in C666-1 overexpressing IL-6R (Figure 5C). *In vivo*, the IL-6R-overexpressing C666-1 cells also grew at a faster rate than control C666-1 cells (Figure 5D). The sizes of tumor xenografts harvested from IL-6R-expressing C666-1 were on average 64% larger than the tumor xenografts established from control C666-1 cells (Figure 5D). These observations showed that IL-6R overexpression enhanced *in vivo* tumorigenicity of EBV-infected NPC cells.

### Overexpression of IL-6R detected in clinical specimens of NPC

**Figure 6:**
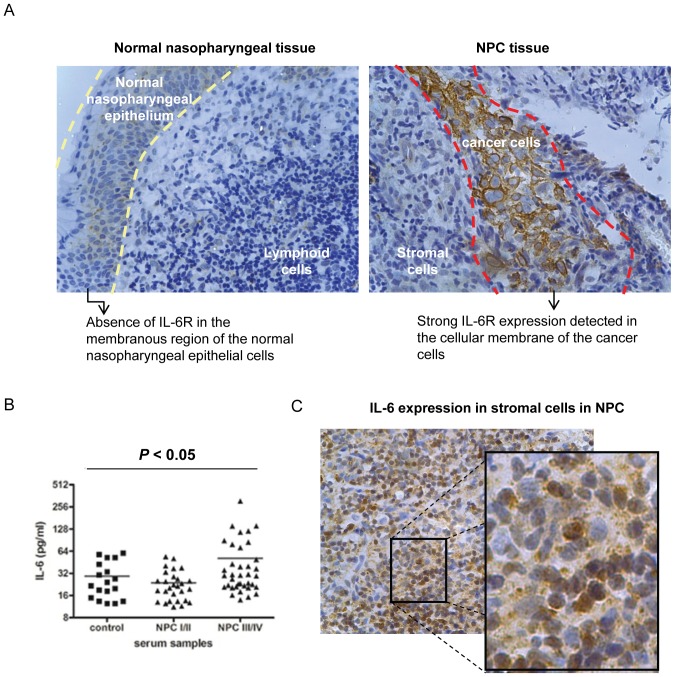
Detection of IL-6R and IL-6 in clinical specimens from NPC patients and control subjects. (A) Strong expression of IL-6R was found in the cancer cells of the NPC tissues but not in the normal nasopharyngeal epithelia. Tissue microarrays (TMAs) of 52 clinical specimens were analyzed for IL-6R by immunohistochemical staining. Representative image showing the expression of IL-6R in normal nasopharyngeal epithelium and NPC cancer cells was shown in the left and right panel respectively. A normal nasopharyngeal epithelium is enclosed by the two dotted yellow lines (in the left image). Underneath the normal nasopharyngeal epithelium are the associated lymphoid cells. A cluster of cancer cells is surrounded by the red dotted lines (in the right images). NPC is highly infiltrated with stromal cells. (B) The levels of IL-6 in sera measured by ELISA from NPC patients were significantly higher than the healthy controls (* *p*<0.05). (C) Representative micrograph showing the expression of IL-6 in the stromal cells of a NPC tissue. An enlarged image of the enclosed area by black rectangle is shown aside.

It has been previously reported that constitutive activation of STAT3 could be detected in more than 70% of NPC cases [8], [9]. We hypothesized that IL-6R expression in NPC cells may be involved in the activation of STAT3 *in vivo*. We therefore examined the expression of IL-6R in NPC tumor biopsies using a tissue microarray (TMA) containing 7 normal nasopharyngeal tissues and 45 NPC specimens. IL-6R is overexpressed in NPC tissues and was detected in 28 out of 45 (62%) NPC specimens examined, while IL-6R expression was not expressed in any of the normal nasopharyngeal tissues examined (0 out of 7 cases; 0%) (Fig 6A). Hence, IL-6R is significantly overexpressed in NPC but not in normal nasopharyngeal tissues (*p*<*0.05*). The IL-6R expression was mainly localized to NPC cells, but not in normal NPE cells and lymphoid infiltrates (Fig 6A). However, when we tried to correlate the IL-6R overexpression with the clinical staging of NPC (i.e stage I, II, III and IV), the sample size was too small for statistical analysis and a larger study is required to fully evaluate the pathological significance of IL-6R in NPC. Since IL-6 is the major inflammatory cytokine that can provoke the IL-6R-mediated STAT3 activation, we compared the levels of IL-6 in the sera of NPC patients and healthy subjects using ELISA. A significant upregulation of the circulating IL-6 was found in the cancer patients, particularly in the advanced-staged individuals, than the healthy controls (Fig 6B). We have also detected the IL-6 expression using NPC tissues by immunohistochemical staining. Strong expression of IL-6 was observed in stromal lymphoid cells (Fig 6C), suggesting that the stromal infiltrate is a rich source of IL-6 in NPC. Taken together, an intricate interaction of IL-6 secretion in stromal cells and overexpression of IL-6R in EBV-infected NPC cells may contribute to the progression of NPC through enhanced activation of STAT3.

## Discussion

EBV infection is closely associated with NPC. However, the exact role of EBV in NPC pathogenesis remains enigmatic. In EBV-infected NPC cells, the EBV episomes are readily lost from the cells upon prolonged culture [5]. We have examined growth rates in EBV-infected primary and immortalized NPE cells as well as NPC cells and failed to observe any increase in growth rate of these cells after EBV infection [24], [25]. In fact, many of our EBV-infected cells exhibited a slower growth rate after infection, which may reflect a cellular response to stress induced by viral infection [3]. Hence, EBV infection *per se* does not appear to confer growth advantage to infected epithelial cells. This observation is counterintuitive as EBV infection is present in most if not all undifferentiated NPC cells *in vivo,* clearly indicating an advantage for NPC cells to retain EBV *in vivo*. A strong selection pressure presumably presents *in vivo* to select EBV-infected NPC cells. The nature of this selective pressure is poorly defined but is believed to be pro-survival and anti-apoptotic in nature. The NPC stroma is rich in inflammatory cells [32]. They may represent potent source of the inflammatory cytokines which affect the survival and malignant properties of the EBV-infected premalignant NPE cells and malignant NPC cells. In this study, we observed IL-6 induced a much more robust STAT3 activation in EBV-infected NPE cells than uninfected cells (Fig 1). The STAT3 activation by IL-6 is well-documented to support growth, survival and metastasis of human cancer cells [14], [16]. *In vitro*, this enhanced cellular response to IL-6-induced STAT3 activation may be beneficial to immortalized NPE cells to overcome the stress-induced growth inhibition commonly associated with EBV infection [23], [25]. In our immortalized NPE cell models, overexpression of IL-6R was found to be primarily responsible for the enhanced response to IL6-activation of STAT3 signaling (Fig 2). Overexpression of IL-6R was shown to confer growth advantage to premalignant NPE cells and NPC cells (Fig 5). Our results also showed that STAT3 activation *per se* enhanced anchorage-independent growth and invasive properties of EBV-infected NPE cells (Fig 3E and 3C). The positive feedback loop of STAT3/LMP1/IL-6/STAT3 may be involved in the pathogenesis and progression of NPC in patients (Fig 4).

The role of chronic inflammation in human carcinogenesis has been long recognized and STAT3 activation is known to be involved [12]. Infection with *H.pylori*, which is closely associated with gastric cancer, also activates STAT3 through its cytotoxin-associated gene A [33]. STAT3 activation is common in NPC [8]–[10]. EBV infection and the expression of viral antigen may also invoke inflammatory response in NPC which stimulates further release of inflammatory cytokines to enhance the inflammatory responses in the host stroma. In this study, we found that the circulating concentration of IL-6 was significantly upregulated in the sera of the advance-staged NPC patients than the control subjects (Fig 6B). Besides, IL-6 expression could be detected in the stromal cells of NPC (Fig 6C). IL-6R expression may confer a selective advantage to EBV-infected NPE cells *in vivo* because of their enhanced response to IL-6-induced STAT3 activation, and therefore cells with higher IL-6R expression are favorably selected EBV-infected NPC cells. This is supported by the observation that IL-6R overexpression in EBV-positive NPC cells, C666-1, enhanced its tumorigenic growth in athymic nude mice (Fig 5D). The positive selection of IL-6R expression in EBV-infected cells may represent an important process in NPC pathogenesis. We have also examined the clinical relevance of IL-6R overexpression in EBV-infected NPC cells in clinical specimens (Fig 6A). IL-6R overexpression can be detected in the NPC cells (28/45 of cases) while the expression of IL-6R is relatively low or absent in normal nasopharyngeal epithelia (Fig 6A). Hence the EBV-infected NPE cells may co-evolve with the host inflammatory stroma during NPC pathogenesis to potentiate the growth and malignant properties of NPC through STAT3 hyperactivation. We could observe overexpression of IL-6R and p-STAT3 in NPC specimens using immunocytochemistry. However, p-STAT3 could also be detected in tissues without IL-6R expression suggesting that multiple pathways are involved in the activation of STAT3 in NPC. The contribution of the EBV-encoded LMP1 to the activation of STAT3 was not evaluated in this study. An earlier study has examined the relationship of LMP1 expression and STAT3 activation in nasopharyngeal carcinoma using immunocytochemistry but failed to reveal a positive correlation of LMP1 expression and STAT3 activation [8]. This finding is not unexpected given the multiple pathways involved in the activation of STAT3 in NPC. In general, LMP1 expression is infrequently detected in NPC by immunocytochemical method which may be related to the low sensitivity of antibody used to detect LMP1 expression in pathological specimens. Using a highly sensitive nested PCR method, LMP1 transcripts were readily detected in most cases of LMP1 [3]. A large scale study with well-controlled NPC specimens collected from different clinical and histopathological stages and the use of sensitive antibody for LMP1 will be required to define the relationship of STAT3 activation with LMP1 and IL-6R expression in EBV-infected NPC and their impact on the clinico-pathological properties of NPC in patients.

In conclusion, we reported in this study that EBV infection in NPE cells enhanced their responsiveness to STAT3 activation by IL-6. Upregulation of IL-6R is involved in the enhanced response of EBV-infected cells to IL-6. STAT3 activation potentiates growth and malignant properties in EBV-infected cells, and also provides a selective advantage for survival of EBV-infected NPC cells *in vivo*. An intricate interaction between EBV infection and stromal inflammatory environment is postulated to be involved in the pathogenesis of NPC and its progression.

## References

[1] Young LS, Rickinson AB (2004) Epstein-Barr virus: 40 years on. Nature reviews Cancer 4: 757–768. 15510157 10.1038/nrc1452

[2] Raab-Traub N (1992) Epstein-Barr virus and nasopharyngeal carcinoma. Seminars in cancer biology 3: 297–307. 1335793

[3] Brooks L, Yao QY, Rickinson AB, Young LS (1992) Epstein-Barr virus latent gene transcription in nasopharyngeal carcinoma cells: coexpression of EBNA1, LMP1, and LMP2 transcripts. Journal of virology 66: 2689–2697. 1313894 10.1128/jvi.66.5.2689-2697.1992 PMC241023

[4] Lo KW, To KF, Huang DP (2004) Focus on nasopharyngeal carcinoma. Cancer cell 5: 423–428. 15144950 10.1016/s1535-6108(04)00119-9

[5] Tsao SW, Tsang CM, Pang PS, Zhang G, Chen H, et al (2012) The biology of EBV infection in human epithelial cells. Seminars in cancer biology 22: 137–143. 22497025 10.1016/j.semcancer.2012.02.004

[6] Man CH, Wei-Man Lun S, Wai-Ying Hui J, To KF, Choy KW, et al (2012) Inhibition of NOTCH3 signalling significantly enhances sensitivity to cisplatin in EBV-associated nasopharyngeal carcinoma. The Journal of pathology 226: 471–481. 22009689 10.1002/path.2997

[7] Buettner R, Mora LB, Jove R (2002) Activated STAT signaling in human tumors provides novel molecular targets for therapeutic intervention. Clinical cancer research : an official journal of the American Association for Cancer Research 8: 945–954. 11948098

[8] Buettner M, Heussinger N, Niedobitek G (2006) Expression of Epstein-Barr virus (EBV)-encoded latent membrane proteins and STAT3 activation in nasopharyngeal carcinoma. Virchows Archiv : an international journal of pathology 449: 513–519. 17033798 10.1007/s00428-006-0294-2

[9] Liu YP, Tan YN, Wang ZL, Zeng L, Lu ZX, et al (2008) Phosphorylation and nuclear translocation of STAT3 regulated by the Epstein-Barr virus latent membrane protein 1 in nasopharyngeal carcinoma. International journal of molecular medicine 21: 153–162. 18204781

[10] Lui VW, Wong EY, Ho Y, Hong B, Wong SC, et al (2009) STAT3 activation contributes directly to Epstein-Barr virus-mediated invasiveness of nasopharyngeal cancer cells in vitro. International journal of cancer Journal international du cancer 125: 1884–1893. 19588483 10.1002/ijc.24567

[11] Bromberg J, Darnell JE Jr (2000) The role of STATs in transcriptional control and their impact on cellular function. Oncogene 19: 2468–2473. 10851045 10.1038/sj.onc.1203476

[12] Yu H, Pardoll D, Jove R (2009) STATs in cancer inflammation and immunity: a leading role for STAT3. Nature reviews Cancer 9: 798–809. 19851315 10.1038/nrc2734 PMC4856025

[13] Lesina M, Kurkowski MU, Ludes K, Rose-John S, Treiber M, et al (2011) Stat3/Socs3 activation by IL-6 transsignaling promotes progression of pancreatic intraepithelial neoplasia and development of pancreatic cancer. Cancer cell 19: 456–469. 21481788 10.1016/j.ccr.2011.03.009

[14] Hirano T, Ishihara K, Hibi M (2000) Roles of STAT3 in mediating the cell growth, differentiation and survival signals relayed through the IL-6 family of cytokine receptors. Oncogene 19: 2548–2556. 10851053 10.1038/sj.onc.1203551

[15] Hodge DR, Hurt EM, Farrar WL (2005) The role of IL-6 and STAT3 in inflammation and cancer. European journal of cancer 41: 2502–2512. 16199153 10.1016/j.ejca.2005.08.016

[16] Bromberg JF, Wrzeszczynska MH, Devgan G, Zhao Y, Pestell RG, et al (1999) Stat3 as an oncogene. Cell 98: 295–303. 10458605 10.1016/s0092-8674(00)81959-5

[17] Darnell JE (2005) Validating Stat3 in cancer therapy. Nature medicine 11: 595–596. 10.1038/nm0605-595 15937466

[18] Lui VW, Yau DM, Wong EY, Ng YK, Lau CP, et al (2009) Cucurbitacin I elicits anoikis sensitization, inhibits cellular invasion and in vivo tumor formation ability of nasopharyngeal carcinoma cells. Carcinogenesis 30: 2085–2094. 19843642 10.1093/carcin/bgp253

[19] Ma N, Kawanishi M, Hiraku Y, Murata M, Huang GW, et al (2008) Reactive nitrogen species-dependent DNA damage in EBV-associated nasopharyngeal carcinoma: the relation to STAT3 activation and EGFR expression. International journal of cancer Journal international du cancer 122: 2517–2525. 18307254 10.1002/ijc.23415

[20] Lo AK, Lo KW, Tsao SW, Wong HL, Hui JW, et al (2006) Epstein-Barr virus infection alters cellular signal cascades in human nasopharyngeal epithelial cells. Neoplasia 8: 173–180. 16611410 10.1593/neo.05625 PMC1578522

[21] Chen H, Lee JM, Zong Y, Borowitz M, Ng MH, et al (2001) Linkage between STAT regulation and Epstein-Barr virus gene expression in tumors. Journal of virology 75: 2929–2937. 11222718 10.1128/JVI.75.6.2929-2937.2001 PMC115919

[22] Chen H, Hutt-Fletcher L, Cao L, Hayward SD (2003) A positive autoregulatory loop of LMP1 expression and STAT activation in epithelial cells latently infected with Epstein-Barr virus. Journal of virology 77: 4139–4148. 12634372 10.1128/JVI.77.7.4139-4148.2003 PMC150666

[23] Eliopoulos AG, Stack M, Dawson CW, Kaye KM, Hodgkin L, et al (1997) Epstein-Barr virus-encoded LMP1 and CD40 mediate IL-6 production in epithelial cells via an NF-kappaB pathway involving TNF receptor-associated factors. Oncogene 14: 2899–2916. 9205097 10.1038/sj.onc.1201258

[24] Tsang CM, Zhang G, Seto E, Takada K, Deng W, et al (2010) Epstein-Barr virus infection in immortalized nasopharyngeal epithelial cells: regulation of infection and phenotypic characterization. International journal of cancer Journal international du cancer 127: 1570–1583. 20091869 10.1002/ijc.25173

[25] Tsang CM, Yip YL, Lo AK, Deng W, To KF, et al (2012) Cyclin D1 supports stable EBV infection in nasopharyngeal epithelial cells. . Proc Natl Acad Sci U S A. 109: 3473–3482. 10.1073/pnas.1202637109 PMC3528537 23161911

[26] Li HM, Man C, Jin Y, Deng W, Yip YL, et al (2006) Molecular and cytogenetic changes involved in the immortalization of nasopharyngeal epithelial cells by telomerase. International journal of cancer Journal international du cancer 119: 1567–1576. 16688717 10.1002/ijc.22032

[27] Dechow TN, Pedranzini L, Leitch A, Leslie K, Gerald WL, et al (2004) Requirement of matrix metalloproteinase-9 for the transformation of human mammary epithelial cells by Stat3-C. Proceedings of the National Academy of Sciences of the United States of America 101: 10602–10607. 15249664 10.1073/pnas.0404100101 PMC489981

[28] Matthews V, Schuster B, Schutze S, Bussmeyer I, Ludwig A, et al (2003) Cellular cholesterol depletion triggers shedding of the human interleukin-6 receptor by ADAM10 and ADAM17 (TACE). J Biol Chem 278: 38829–38839. 12832423 10.1074/jbc.M210584200

[29] Lo AK, Liu Y, Wang XH, Huang DP, Yuen PW, et al (2003) Alterations of biologic properties and gene expression in nasopharyngeal epithelial cells by the Epstein-Barr virus-encoded latent membrane protein 1. Laboratory investigation; a journal of technical methods and pathology 83: 697–709. 12746479 10.1097/01.lab.0000067480.44925.10

[30] Albini A, Iwamoto Y, Kleinman HK, Martin GR, Aaronson SA, et al (1987) A rapid in vitro assay for quantitating the invasive potential of tumor cells. Cancer research 47: 3239–3245. 2438036

[31] Euhus DM, Hudd C, LaRegina MC, Johnson FE (1986) Tumor measurement in the nude mouse. J Surg Oncol 31: 229–234. 3724177 10.1002/jso.2930310402

[32] Gourzones C, Barjon C, Busson P (2012) Host-tumor interactions in nasopharyngeal carcinomas. Seminars in cancer biology 22: 127–136. 22249142 10.1016/j.semcancer.2012.01.002

[33] Bronte-Tinkew DM, Terebiznik M, Franco A, Ang M, Ahn D, et al (2009) Helicobacter pylori cytotoxin-associated gene A activates the signal transducer and activator of transcription 3 pathway in vitro and in vivo. Cancer research 69: 632–639. 19147578 10.1158/0008-5472.CAN-08-1191 PMC3418672

